# A Formulation–Process–Product Integrated Design Method for Accelerating Pharmaceutical Tablet Development via the High-Shear Wet Granulation and Tableting Route

**DOI:** 10.3390/pharmaceutics17030322

**Published:** 2025-03-02

**Authors:** Zichen Liang, Xuefang Tang, Liping Chen, Yifei Liu, Shuying Zhao, Xiao Ma, Gan Luo, Bing Xu

**Affiliations:** 1 Department of Chinese Medicine Informatics, Beijing University of Chinese Medicine, Beijing 100029, China; liangzichen0401@163.com (Z.L.); 15737010383@163.com (X.T.); 20230935129@bucm.edu.cn (L.C.); liudongdexiaowu@163.com (Y.L.); bucmzsy@163.com (S.Z.); bucmmx@163.com (X.M.); 2 Beijing Key Laboratory of Chinese Medicine Manufacturing Process Control and Quality Evaluation, Beijing 100029, China

**Keywords:** high-shear wet granulation and tableting (HSWGT), material library, quality by digital design (QbDD), formulation-process-product integrated design (FPPID), in silico simulation

## Abstract

**Background/Objectives**: Tablet is the most popular oral solid dosage form, and high-shear wet granulation and tableting (HSWGT) is a versatile technique for manufacturing tablets. The conventional pharmaceutical development for HSWGT is carried out in a step-by-step mode, which is inefficient and may result in local optimal solutions. Inspired by the co-design philosophy, a formulation–process–product integrated design (FPPID) framework is innovatively brought forward to enable the target-oriented and simultaneous exploration of the formulation design space and the process design space. **Methods**: A combination of strategies, such as a material library, model-driven design (MDD), and simulation-supported solution generation, are used to manage the complexity of the multi-step development processes of HSWGT. The process model was developed at the intermediate level by incorporating dimensionless parameters from the wet granulation regime map approach into the process of the partial least square (PLS) model. The tablets tensile strength (*TS*) and solid fraction (*SF*) could be predicted from the starting materials’ properties and process parameters. The material library was used to diversify the model input and improve the model’s generalization ability. Furtherly, the mixture properties calculation model and the process model were interconnected. **Results**: A four-step FPPID methodology including the target definition, the formulation simulation, the process simulation, and the solution generation was implemented. The performance of FPPID was demonstrated through the efficient development of high-drug-loading tablets. **Conclusions**: As a holistic design method, the proposed FPPID offers great opportunity for designers to handle the complex interplay in the sequential development stages, facilitate instant decisions, and accelerate product development.

## 1. Introduction

Tablet is the most frequently used oral solid dosage (OSD) form for clinical use, and it has the advantages of allowing for an accurate dosage, portability, and ease of administration. According to the report on new drug approvals from the Center for Drug Evaluation and Research (CDER) in the US Food and Drug Administration (FDA), OSD formed accounted for 40.0% of all new drugs approved in 2023 and tablets accounted for 68.2% of the total number of OSD forms [1]. The Manufacturing Classification System (MCS) working group partitioned the processing routes of tablets into four types, i.e., direct compression (DC), dry granulation (DG), wet granulation (WG), and others [2]. Among them, the WG processing routes are complex and time-consuming. This technique is not suitable for moisture-sensitive compounds [3]. Also, there is a risk of solvent residue remaining after wet granulation [4]. However, it can deal with a wider range of material properties, as well as improving material’s flowability, uniformity, and tableting capacity [5]. At the same time, WG can reduce the generation of dust in the production process and reduce the difficulty of cleaning the equipment. DC and DG processes have higher requirements for the properties of the material itself. When the flowability of the material is very poor and the conditions for using DC and DG processes are not met, WG is a good choice [6]. In addition, when the dosage of the Active Pharmaceutical Ingredient (API) is low and needs to be dispersed [7], wet granulation and tableting obtain better quality. The benchmarking industrial application of wet granulation is the high-shear wet granulation method, which has many advantages, like a short processing time, quick production changeover, and good process reproducibility [8]. It can handle compounds with high viscosity [9]. Therefore, the high-shear wet granulation process was selected for this study.

High-shear wet granulation and tableting (HSWGT) involves a series of processing units such as powder-mixing, granulation, drying, milling, lubrication, and tableting. The initial and intermediate material properties and process parameters interact with each other to influence the final tablet’s performance [10]. The understanding of the processes of HSWGT could be deepened using the Quality by Design (QbD) approach. On the basis of clarifying the quality target product profile and identifying the critical quality attributes, the formulation development and the optimization of each manufacturing unit were carried out step-by-step mode [11]. That is, only after the design, optimization, and validation of one stage was the development transferred to the next stage [12]. The main reason for adopting the step-by-step mode is that the knowledge of the whole development process is not fully understood. In the absence of prior knowledge, the step-by-step mode provided a footpath along which the knowledge of the process, design, and accumulated expertise, as well as the scientific development, could reliably progress. However, this unit-by-unit optimization may result in local optimum solutions, the interactions between each step may be neglected, and the related experimental tests are time-consuming and expensive. An alternative and promising approach is model-driven design (MDD) or model-based design (MBD) for integrated processes [13,14,15], where the design space exploration is driven by in silico simulations instead of extensive experiments, and the unit optimization is realized as part of the entire process. For instance, Boukouvala et al. [16] pioneered a dynamic flowsheet model to simulate a tablet manufacturing process that involved multiple processing steps, such as powder feeding, blending, wet granulation, drying, milling, and tableting. It was found that critical operating conditions such as the liquid binder addition rate and the milling speed strongly affected the hardness and dissolution of the produced tablets. In another example, Metta et al. [17] implemented a simplified flowsheet model using a wet granulation manufacturing route, including feeders, mixers, TSWG, a fluid bed dryer, a mill, and a tablet press. Various modeling approaches (e.g., empirical, semi-empirical, statistical, and mechanistic) were combined to establish a system model. The model allows for an understanding of the effect of changes in five process variables (i.e., flow rate, liquid to solid ratio, granulator screw speed, dryer air temperature, and drying time) on the final tablet’s properties (i.e., tablet hardness). The above works only focused on the design and optimization of the entire process for a single fixed-formulation product, like the acetaminophen tablet, and did not consider the formulation optimization step.

Generally, tablet formulation design is a challenging task due to the complex physical interactions between powdered ingredients under various processing conditions. Some recent studies used the material library approach to establish a database of the physical properties of relevant drugs and excipients [18,19,20,21]. After assessing the performance of selected single materials for a given processing route, a universal knowledge space or design space linking the properties of various materials, the process parameters, and the tablet quality attributes could be built. The quality attributes of new multicomponent tablets can then be predicted from the formulation mixture properties that were estimated by mixing models [22] or by considering the percolation threshold [23]. Recently, the material library approach has been successfully applied to several pharmaceutical development scenarios, such as direct compression [24], continuous powder feeding and blending [25], dry granulation and tableting [17], etc. As for wet granulation and tableting, Wang et al. [21] used the material database and data fusion method to develop a formulation–process–quality model of the HSWG process. Nevertheless, only the granule sizes were predicted and tuned. Furtherly, Wang Y.W. et al. [20] established a material library containing 30 pharmaceutical materials, mainly investigating the effects of material properties on the tensile strength (*TS*) and solid fraction (*SF*) of tablets prepared using the HSWGT. In order to simplify the experimental design, the process parameters of wet granulation were fixed without considering the synergistic effect of material properties and process variables on the tablets. However, the above studies have the common shortcoming that formulation variables and process variables were not studied simultaneously.

Inspired by the co-design philosophy and concurrent engineering in the manufacturing industry [26,27], a formulation–process–product integrated design (FPPID) framework is innovatively brought forward in this paper to accelerate tablet development through the high-shear wet granulation and tableting route. With the increasing application of digital technology in pharmaceutical development, the extensively used QbD paradigm has gradually evolved into quality by digital design (QbDD), which is a computational model-aided enhancement of the QbD framework. Combinatorial strategies such as the state-of-the-art material database approach, model-driven design, and simulation-supported solution generation are used to manage the complexity of the multi-step development processes of HSWGT in this paper. As a holistic design method, the FPPID aims to help designers (i) integrate domain knowledge from diverse sources and thoroughly understand the important connections in a system constituting materials, manufacturing processes, and tablet products, (ii) identify reliable formulation and process solutions in the high-dimensional design space to ensure both targeted tablet performance and overall system performance, and (iii) reduce the dependency on time-consuming and expensive laboratory-scale experiments. The rest of the paper is arranged as follows. In Section 2, the material-sparing characterization approach for selecting representative powders is introduced. A customized experimental design and the experimentation methods of the HSWGT processes for generating organized data involving material properties and process parameters are detailed. Then, the latent variable model for data-driven process modeling is presented. Section 3 presents the results and discussions of the study. The effectiveness of the material-sparing approach is demonstrated. The predictive modeling and an associated sensitivity analysis are carried out. The FPPID procedures are addressed, and the applicability of the proposed mythology is showcased. Finally, Section 4 concludes and makes recommendations for future research.

## 2. Materials and Methods

### 2.1. Materials

**Figure 1:**
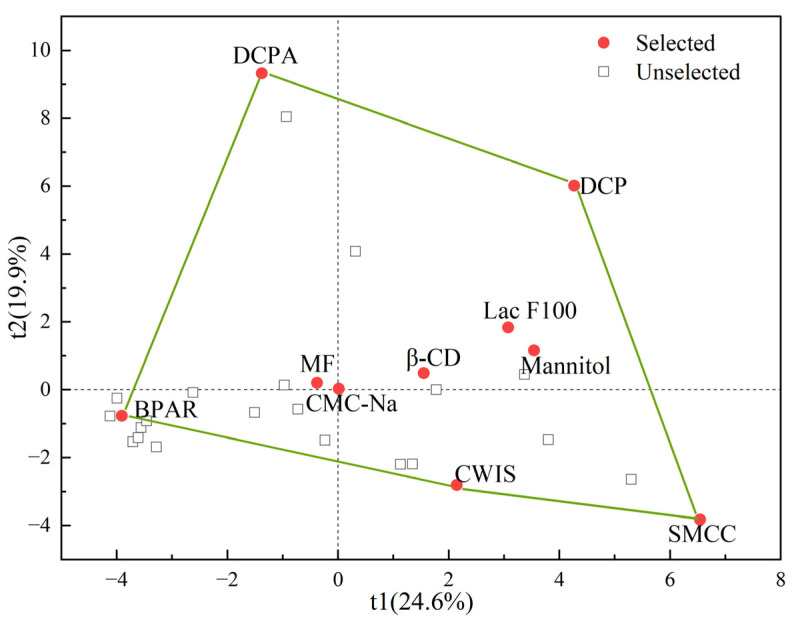
The selection of representative materials based on the PCA model from the homemade material library. DCPA, dibasic calcium phosphate anhydrous; DCP, dibasic calcium phosphate; SMCC, silicified microcrystalline cellulose; CWIS, cold-water-insoluble starch; BPAR, Bran-Processed Atractylodis Rhizoma; CMC-Na, croscarmellose sodium; MF, Mume Fructus; Lac F100, Lactose Flowlac^®^ 100; β-CD, β-cyclodextrin. The red dots are selected materials. The hollow gray squares are unselected materials. The green line area represents the basic distribution of the material’s physical properties in the PCA scoring plot.

With the help of the material-sparing characterization method [28] and principle component analysis, a representative material library containing 10 pharmaceutical materials was built. The data source for selecting materials is a homemade material library with a total of 30 material property data records [20]. Firstly, the principal component analysis was carried out on the primary material dataset, where each material featured 19 physical property parameters, 9 compression behavior classification system (CBCS) parameters, and 2 tabletability change indexes (i.e., the reworking potential and the relative change in tabletability). The first and second principal components were found to explain 24.6% and 19.9% of the total variation. Then, the selection was carried out by considering five boundary points in the score space, as shown in Figure 1, in order to maximize the information coverage. The chosen five boundary point materials were dibasic calcium phosphate anhydrous (DCPA, Lot No. D03424A, TechTex-Verlag GmbH & Co. KG, Budenheim, Germany), dibasic calcium phosphate (DCP, Lot No. C93226A, TechTex-Verlag GmbH & Co. KG, Budenheim, Germany), silicified microcrystalline cellulose (SMCC, Lot No. ZC20501, SPI Pharmaceutical Technology Co., Ltd., Wilmington, NC, USA), cold-water-insoluble starch (CWIS, Lot No. Y26S10F98934, Shanghai Yuanye Bio-Technology Co., Ltd., Shanghai, China), and Bran-Processed Atractylodis Rhizoma extract powders (BPAR, Lot No. PYP180209-067400-07A, Beijing Tcmages Pharmaceutical Co., Ltd., Beijing, China). The croscarmellose sodium (CMC-Na, Lot No. 1098NXX, DFE Pharma GmbH & Co. KG, Goch, Germany) and the Mume Fructus extract powders (MF, Lot No. J180606-672600-07A, Beijing Tcmages Pharmaceutical Co., Ltd., Beijing, China) were selected as centerpoint materials. In addition, considering the uniform distribution of materials in the latent space, three materials in the score plot were selected, namely Lactose Flowlac^®^ 100 (Lac F100, Lot No. L101501320, Molkerei Meggle Wasserburg GmBH & Co. KG, Wasserburg, Germany), β-cyclodextrin (β-CD, Lot No. 200311, Liaoning Luquan Pharmaceutical Technology Co., Ltd., Shenyang, China) and mannitol (Lot No. 122000024, SPI Pharmaceutical Technology Co., Ltd., Wilmington, NC, USA). The selected 10 materials included eight pharmaceutical excipients and two natural product powders (NPPs).

The natural product powder, i.e., the Menthae Haplocalycis Herba extract powders (MHH, J180627-611300-13A, Beijing Tcmages Pharmaceutical Co., Ltd., Beijing, China), was used to mimic the high-drug-loading formulations to be designed.

### 2.2. The Design of Experiment

In order to develop a generalized data-driven model for the HSWGT process and to explore the effect of HSWGT process parameters on the tensile strength (*TS*) and solid fraction (*SF*) of tablets, a hybrid experimental design was used to arrange both the material type factor and key process factors. For the high-shear wet granulation process, a customized experimental design, with the help of the Design Expert software 13.0 (Stat-Ease, Minneapolis, MN, USA), was used to arrange four factors, i.e., the type of input material, the liquid to solid (*L*/*S*) ratio, the impeller speed, and the wet massing time (*WT*), as shown in Table 1. The type of input material is considered separately as a blocking factor. The experimental runs are determined using the D-optimal selection criterion during the build, and 30 experimental runs were arranged, as shown in Table S1 of Supplementary Material S1. For the tableting process, the tableting pressure (*P*) was viewed as the process factor to be studied. A tableting force varying from 1 kN to 11 kN at an interval of 2 kN was combined with each run in the customized design presented above. Therefore, a total of 180 experimental runs were generated for the tablet preparation.

The material library was used to diversify the input of the following process model. In Table S1 of Supplementary Material S1, the 30 experimental runs were split into 10 blocks, and 10 materials in Section 2.1 were randomly assigned to different blocks. To avoid introducing any additional material, the deionized water and 95% (*v*/*v*) alcohol were used as wetting agents. The 95% (*v*/*v*) alcohol was applied when the water wet materials produced sticky wet mass or hard granules. Since the type of wetting agent was specific to the material, this factor was not considered to be varied in the experimental design.

The ranges of the *L*/*S* ratio differed from material to material and were ascertained by preliminary experiments. The minimum amount of wetting agent to be added was just enough to pass the “fist test” [29], in which the operator squeezed a sample of the wet mass and judged the proper consistency of wet mass with less fine powder. The maximum amount of wetting agent was determined when the wet mass formed a slurry. In the experimental design, the *L*/*S* ratio was treated as a categorical factor, and three levels, including the high, the middle, and the low, were varied. For a specific material, the high and low levels corresponded to the maximum and minimum amount of wetting agent, respectively.

The impeller speed and the wet massing time were two numerical factors. The impeller speeds were designed to be varied from 400 rpm to 800 rpm. Within this range, both the pharmaceutical excipient and the natural product powder can be sufficiently mixed with the wetting agent to form granules. After the addition of the wetting agent, the wet massing times were designed to be in the range of 180 s to 300 s, within which the granules were successfully made.

### 2.3. Preparation of Granules and Tablets

#### 2.3.1. The High Shear Wet Granulation Process

For each run in Table S1 of the Supplementary Material S1, about 300 g of powders were poured into a high-shear granulator (SHK-4, Xi’an Runtian Pharmaceutical Machinery Co., Ltd., Xi’an, China) with a 2 L granulation pot. Before granulation, dry mixing was carried out for 60 s at the impeller speed of 300 rpm without switching on the chopper. Then, the granulator was run at an impeller speed specified in Table S1 of the Supplementary Material S1. The chopper speed was kept constant at 1600 rpm in all experiments. After that, the wetting agent was added using a peristaltic pump (LabV1, Baoding Shenchen Pump Co., Ltd., Baoding, China). The addition time of the wetting agent was kept constant at 180 s in all cases. At the end of the granulation process, the wet mass was removed to pass through a 10-mesh standard sieve and spread out on a tray and dried in an oven (DHG-9030, Shanghai Yihang Scientific Instrument Co., Ltd., Shanghai, China) at 55 °C for 12 h. The dry granules were sieved using a vibrator screener (ZNS-300, Beijing Xingshi Li-He Technology Development Co., Ltd., Beijing, China) and granules with size segments of 125–250 μm were used in the subsequent tableting process. The purpose of selecting granules of the same size was to exclude the impact of granule sizes on tablet quality, as well as to facilitate the comparison of the physical properties of granules made from different materials.

Two dimensionless parameters, i.e., the Froude number (*Fr*) and the maximum pore saturation (${S^{\prime }}_{\max}$), were introduced to describe the process characteristics. The Froude number (*Fr*) indicates the agitation intensity of the granulation process [30,31] and is calculated as follows:

(1) $$\mathrm{Fr}=\frac{\omega^{2}(2R)}{g}$$

*R* in the equation indicates the inner diameter (m) of the granulation pot. ω denotes the number of revolutions per second (rps) of the impeller. *g* denotes the acceleration of gravity.

The maximum pore saturation [32] indicates the maximum extent to which voids in a material are filled with fluid and reflects the environment determining the growth behavior of granules. ${S^{\prime }}_{\max}$ is the ratio of granule liquid volume to granule void volume and the formula is as follows:

(2a) $${S^{\prime }}_{\max}=\frac{w\rho_{s}(1-\varepsilon_{\min})}{\rho_{l}\varepsilon_{\min}}$$

(2b) $$\varepsilon_{\min}=1-\frac{\rho_{e}}{\rho_{p}}$$

In the above equation, *w* is the mass ratio of liquid to solid. $\rho_{s}$ is the bulk density of solid granules. $\rho_{l}$ is the liquid density. $\varepsilon_{\min}$ is the minimum porosity that can be achieved by the prescription under the given operating conditions. $\rho_{p}$ is the true density of the powder and $\rho_{e}$ is the envelope density of the powder. Nevertheless, due to the fact that the envelope density was unknown, the tapped density of the material was used instead in this paper. Therefore, the ${S^{\prime }}_{\max}$ denotes the dry-tapped porosity that is filled with fluid.

#### 2.3.2. The Tableting Process

The granules were pressed under six different pressure conditions (i.e., 1 ± 0.5, 3 ± 0.5, 5 ± 0.5, 7 ± 0.5, 9 ± 0.5 and 11 ± 0.5 kN, 1 kN = 12.74 MPa) using a single-punch tablet press machine (C&C600A, Beijing Chuangbo Jiawei Co., Ltd., Beijing, China), which was equipped with the flat-faced punch and die of 10 mm in diameter. At least four tablets were available for each pressure condition. The tablet weight was adjusted on the basis of powders’ bulk density. The powders with a bulk density greater than 0.35 g·mL^−1^ was weighed as 0.35 g and the powders with a bulk density less than 0.35 g·mL^−1^ were weighed as 0.30 g. The magnesium stearate was applied to lubricate the punch and the inner wall of the die before each compaction. The powders or granules were filled into the die via manual filling. The compression pressure was recorded when the minimum separation distance between the upper and lower punches was reached. The obtained tablets were sealed in plastic bags and were stored for 72 h at room temperature. The mass of tablets was determined using an analytical balance (GL124-1SCN, Sanfuhezhong (Beijing) Science and Technology Development Co., Ltd., Beijing, China). The thickness and diameter of the tablets were measured using a digital caliper (547-401 Digimatic Caliper, Mitutoyo, Kawasaki, Japan). The hardness of tablets was measured using an intelligent tablet hardness tester (YPD-500C, Shanghai Huanghai Pharmaceutical Inspection Instrument Co., Shanghai, China). The tensile strength (*TS*) and solid fraction (*SF*) of tablets were calculated using Equation (3) and Equation (4a), respectively.

(3) $$\mathrm{TS}\;=\frac{2F}{\pi \mathrm{DH}}$$

where *F* (N) is the tablet crushing force, *D* (mm) is the tablet diameter, and *H* (mm) is the tablet thickness [33].

(4a) SF=1−ε

(4b) $$\varepsilon \;=\frac{\rho_{\mathrm{app}}}{\rho_{\mathrm{true}}}$$

(4c) $$\rho_{\mathrm{app}}=\frac{m}{\pi \frac{D^{2}}{4}H}$$

where *ε* is the tablet porosity, $\rho_{\mathrm{app}}$ is the apparent tablet porosity, $\rho_{\mathrm{true}}$ (g·cm^−3^) is the true tablet density, which is the same as the true density of the material, and *m* (g) is the tablet weight.

### 2.4. Characterization of Powders

#### 2.4.1. The Physical Properties

According to the material characterization method of the iTCM database established in the previous work [34], 19 physical parameters were measured to describe six incidence factors of granules, including the fundamental property (*D*_t_, *D*_10_, *D*_50_, *D*_90_), the dimension (*D*_a_, *D*_c_, $\varepsilon_{p}$, *SF*_p_), the compressibility (*Ie*, *IC*, *Icd*), the flowability (*IH*, *AOR*, *t*″), the stability (*%HR*, *%H*), and the homogeneity (*%pf*, *Iθ*, *Span*). The acronym and unit of each physical parameter are shown in Table 2.

#### 2.4.2. The Compression Behavior

The compression behavior classification system (CBCS) is an efficient approach for assessing the powder compression behavior in terms of compressibility, compactibility, and tabletability [34,35,36]. Nine descriptors from a range of compression models are included in the CBCS, as shown in Table 3. The *a*, *b*^−1^, and *ab* variables of the Kawakita equation [37], the Heckel *P_y_* [38], the Shapiro *f* [39], and the Gurnham *K* [40] are compressibility descriptors. The Ryshkewitch–Duckworth *k_b_* [41,42] contributes to explain the compactibility. The tabletability is denoted by the parameters *d* and *g* [43] from the Power equation. Each fitted compression equation was evaluated by the coefficient of determinism (*R*^2^) and the root mean square error (*RMSE*).

### 2.5. The Multivariate Data Analysis

Principal component analysis (PCA) was used to perform the dimensionality reduction and to extract the relationships between measurements in the data. The partial least squares (PLS) algorithm was used to correlate the input and output variables and to create a regression model that could be used for prediction. Before modeling, all data were preprocessed by the mean centering and unit variance (UV) scaling in order to eliminate the dimension differences among different variables. The PLS models were internally validated using a seven-fold cross-validation approach. The performance of the model was evaluated through quantitative indexes, such as the cumulative *R*^2^*X* value (*R*^2^*X*_cum_), the cumulative *R*^2^*Y* value (*R*^2^*Y*_cum_), the cumulative *Q*^2^ value (*Q*^2^_cum_), the root mean square error (*RMSE*), and the root mean square error of cross-validation (*RMSECV*). The *R*^2^*X*_cum_ and *R*^2^*Y*_cum_ represent the interpretability of the model, and the larger the *R*^2^*X*_cum_ and *R*^2^*Y*_cum_ values, the more information the model can explain [44]. The *R*^2^*X*_cum_ and *R*^2^*Y*_cum_ are calculated as in Equation (5a) and (5b):

(5a) $$R^{2}X_{\mathrm{cum}}=1-\frac{\sum_{i-1}^{n}\sum_{j-1}^{m}{\left(X_{\mathrm{ij}}-\hat{X_{\mathrm{ij}}}\right)}^{2}}{\sum_{i-1}^{n}\sum_{j-1}^{m}X_{\mathrm{ij}}^{2}}$$

(5b) $$R^{2}Y_{\mathrm{cum}}=1-\frac{\sum_{i-1}^{n}\sum_{k-1}^{p}{\left(Y_{\mathrm{ik}}-\hat{Y_{\mathrm{ik}}}\right)}^{2}}{\sum_{i-1}^{n}\sum_{k-1}^{m}Y_{\mathrm{ik}}^{2}}$$

where X*_ij_* is the value of row *i*, column *j* in the original data. $\hat{X_{ij}}$ is the corresponding value after reconstruction through the PLS model. Y*_ik_* is the actual value of the *k*-th dependent variable for the *i*-th sample. $\hat{Y_{ij}}$ is the predicted value of Y*_ik_* by the model.

The *Q*^2^_cum_ is a metric that assesses the predictive power of a model through cross-validation and reflects the model’s generalization performance with new data. The larger the value of *Q*^2^_cum_, the better the predictive performance of the model. The *Q*^2^_cum_ [45] is calculated as in Equation (5c):

(5c) $${Q^{2}}_{\mathrm{cum}}=1-\frac{{PRESS}_{\mathrm{cum}}}{\mathrm{SS}}$$

where ${PRESS}_{\mathrm{cum}}$ is the cumulative predict residual error sum of squares. The data were divided into seven subsets and the *PRESS* was calculated by training the model with six subsets at a time and predicting using the remaining subset. This was repeated seven times to accumulate all the *PRESS*. *SS* is total sum of squares.

The *RMSE* [46] reflects how well the model fits the known data and is able to quantify the model’s prediction error on the training set. The *RMSE* for the working set can be referred to as the root mean square error of estimation (*RMSEE*), and the *RMSE* for the prediction set can be referred to as the root mean square error of prediction (*RMSEP*). The *RMSE* is calculated as in Equation (5d):

(5d) $$\mathrm{RMSE}=\sqrt{\frac{\sum {\left(Y_{\mathrm{obs}}-Y_{\mathrm{pred}}\right)}^{2}}{N}}$$

where *Y_obs_* represents the true values of samples in the test set and *Y_pred_* represents the predicted value from the model for the test set. *N* represents the number of samples in the test set.

The *RMSECV* [44] measures the model’s prediction error during cross-validation. A smaller value of *RMSECV* usually indicates better model performance. The *RMSECV* is calculated as in Equation (5e):

(5e) $$\mathrm{RMSECV}=\sqrt{\frac{\sum_{i-1}^{n}{\left(y_{i}-{\hat{y}}_{i}\right)}^{2}}{n}}$$

where *y_i_* represents the true value of the *i* sample, ${\hat{y}}_{i}$ represents the predicted value of the *i* sample in cross-validation, and *n* represents the total number of samples.

The structure of the PLS model was controlled by the number of latent variables (LVs), which were fine-tuned using the model evaluation criteria to improve the accuracy of the model. Hotelling T^2^ statistics [47] were used to measure the degree of variability of a sample in the latent variables space and to help identify outlier samples. The limit of the Hotelling T^2^ range is an indicator of outliers. The limit of the Hotelling T^2^ range is calculated as in Equation (5f):

(5f) $$T_{\lim}^{2}=\frac{A(n-1)}{n-A}\times F_{A,\;n-A;\;\alpha }$$

where *n* is the number of training samples and $F_{A,\;n\;-\;A;\;\alpha }$ is the critical value of the *F* distribution with degrees of freedom *A* and *n*−*A* at significance level *α*. Values greater than the 95 percent confidence interval limit are suspect, and values greater than the 99 per cent confidence interval limit can be considered serious abnormal values.

The square prediction error (*SPE*) is a chemometric metric in the PLS model, which is used to measure the degree of abnormality of samples in the model’s residual space [48]. It is commonly used to detect anomalous samples that do not fit the model assumptions. The limit of the SPE range is a metric of abnormal value. The calculation of *SPE* thresholds is generally determined using statistical distributions or empirical methods. The specific Equation (5g) is as follows:

(5g) $$\mathrm{SPE}=\sum_{j=1}^{m}{\left(x_{\mathrm{ij}}-\hat{x_{\mathrm{ij}}}\right)}^{2}$$

where *x_ij_* is the original observation of the *j*-th variable for the *i*-th sample. $\hat{x_{ij}}$ is the predicted value of the *j*-th variable of the PLS model through the latent variable. *m* is the number of original variables.

The multivariate analyses were performed with the help of the SIMCA 13.0 software (Sartorius, Göttingen, Germany). The fitting of the compression equations, as well as the data processing and analysis of the simulations, were performed using MATLAB R2023a (MathWorks, Natick, MA, USA) software.

## 3. Results and Discussion

### 3.1. The Effectiveness of the Material Library Approach

Both the representative materials selection in Section 2.1 and the hybrid design of the experiment in Section 2.2 aimed to provide as much information as possible about the effects of material–process interactions on the tablet products. The richness and diversity of the obtained information were critical to the universality of the design method and could be evaluated by looking at the physical properties and the compression behavior of the resulting intermediate granules. Given the parameters designed in Table S1 of Supplementary Material S1, 30 batches of granules were using the preparation methods presented in Section 2.3.1. The physical properties of the granules were characterized according to Section 2.4. The 30 batches of granules prepared in this paper were compressed into tablets using different compression pressures in the range of 1–11 kN (10–140 MPa) according to the method in Section 2.3.2. For 10 powders and 30 granules, the six compression models specified in Table 3 were fitted. Among the obtained compression equations, 235 out of the 240 fitted equations had *R*^2^ values above 0.9, showing good model fitting. The remaining five equations had *R*^2^ values higher than 0.85, which were acceptable. The material characterization data can be found in Table S2 of Supplementary Material S1.

**Figure 2:**
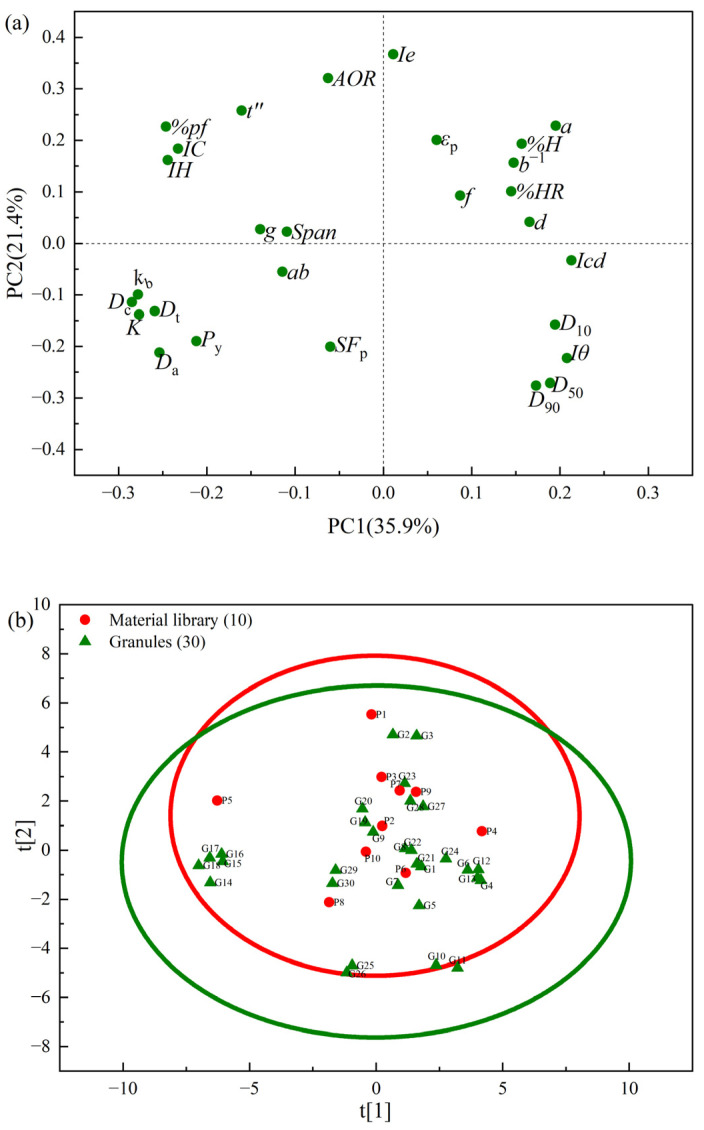
The PCA of the physical properties data based on the first two principal components: (**a**) the loading plot; (**b**) the score plot (The red dots are 10 materials from the material library. The green positive triangles represent the granules).

The matrix **X1** (size 40 × 28), consisting of 19 physical properties and 9 compression descriptors of 40 samples (i.e., 10 powders from the material library and 30 granules), was subjected to principal component analysis. The first two principal components (PCs) were able to explain 57.3% of the total variation in the matrix **X1**. Figure 2a shows the loading plot under PC1 and PC2, which explains 35.9% and 21.4% of the total variation, respectively. In Figure 2a, the physical parameters on the same side of the PC are positively correlated and the opposite variables are negatively correlated. The main physical properties associated with PC1 were the cohesion index (*Icd*) and the compression behavior descriptors (i.e., g, *ab* and *d*). The compression descriptor *g* was located on the negative axis of PC1, opposite to the cohesion index (*Icd*). This result suggests that the enhancement of the material’s cohesive force may reduce its sensitive response to external pressure by improving its structural stability. The solid fraction (*SF*_p_) was located opposite the inter-particle porosity (*Ie*) and the powder porosity ($\varepsilon_{p}$) in PC2. The density parameters (*D*_a_, *D*_c_ and *D*_t_) and the compression descriptors (*k*_b_, *K*) were located in the same quadrant as the stability parameters (*%HR*) and compression descriptor (*d*), in the opposite direction. These results indicate that as the density increased, the compressibility resistance of the material also increased, whereas the bonding capacity between particles may decrease and the tabletability became poorer. It is worth noticing that the compression descriptor (*P*_y_) is located in the opposite direction to the compression parameters (*f*, *a*), and the particle size parameters (*D*_50_, *D*_90_) are located in the opposite direction to the flowability parameter (*t*″). This indicates that the stronger the compression capacity of the granules, the better the plastic deformation capacity, and the more likely they are to be fragmented. Flowability is improved by larger particle sizes.

Figure 2b shows the score plot of the PCA model, where ten powders are represented by red dots and the prepared granules are represented by green positive triangles. The 95% confidence ellipses for powders and granules were drawn in red and green colors, respectively. Compared with the red circle, it seems that the green circle moved downward. This indicates that, after wet granulation, the flowability of the granules becomes better and the inter-particle porosity decreases. The area of the green confidence ellipse was larger than that of the red confidence ellipse, indicating that varying process parameters could result in an expansion of the physical properties of granules, and the diversity of the modeling data would be guaranteed.

### 3.2. The Predictive Modeling of the High-Shear Wet Granulation and Tableting Process

Modeling the integrated high-shear wet granulation and tableting processes is the foundation of model-driven design. In this paper, the final tablets’ quality attributes are predicted from both the starting material properties and the process operations, which are varied in the hybrid experimental design. As shown in Table 4, 19 powder physical properties, 9 powder compression descriptors, 4 HSWG process parameters, and the tableting pressure were taken as input variables. The HSWG process parameters included the *L*/*S* ratio, the wet massing time (*WT*), *Fr*, and ${S^{\prime }}_{\max}$. The output variables of the model were the tensile strength (*TS*) and solid fraction (*SF*). Within each tableting pressure range (e.g., 5 ± 0.5 kN), four tablets were made and the measured compression forces were recorded. For every tablet product, the corresponding powder properties and compression descriptors, as well as the HSWG process parameters, were combined into an input vector. Theoretically, 30 batches of granules compressed under six tableting pressure ranges would generate 720 observations (i.e., 30 × 6 × 4 = 720). The actual number of obtained tablets was 735 due to the increased number of compression tests at certain tableting pressure levels.

Partial least squares regression was carried out to establish a prediction model for the output variables. The latent variables (LVs) controlled the structure of the PLS model. As the number of latent variables increased, the information extracted from the model increased. However, there was a risk of overfitting when the number of LVs was too large. After fine-tuning the number of LVs, the PLS Model M1 with six latent variables was built, and the cumulative *R*^2^*X*, *R*^2^*Y*, and *Q*^2^ of Model M1 were 0.816, 0.785, and 0.781, respectively. Meanwhile, the *RMSE* of model M1 on different predictors was 1.08 for *RMSE*_(TS)_ and 0.05 for *RMSE*_(SF)_. The *RMSECV* of model M1 on different predictors was 1.09 for *RMSECV*_(TS)_ and 0.05 for *RMSECV*_(SF)_. Furthermore, the PLS Model M1 was simplified and optimized by omitting the rearrangement index *ab*, the fragmentation descriptor *f*, and the pressure sensitivity descriptor *g,* which were redundant variables with variable importance for projection (VIP) values less than 1, and a refined PLS Model M2 was established. In addition, in Figure 2b, it can be seen that the omitted three variables are close to the center of the loading plot and have less influence on the modeling results. Under six LVs, the cumulative *R*^2^*X*, *R*^2^*Y*, and *Q*^2^ of the PLS Model M2 were 0.819, 0.786, and 0.782, respectively, suggesting a slightly increased predictive performance. Meanwhile, the *RMSE* values of model M2 on different predictors are 1.09 for *RMSE*_(TS)_ and 0.05 for *RMSE*_(SF)_. The *RMSECV* values of model M2 on different predictors are 1.10 for *RMSECV*_(TS)_ and 0.05 for *RMSECV*_(SF)_. It can be observed that when these three variables are reduced, the changes in model indicators *R*^2^*X*_cum_, *R*^2^*Y*_cum_, and *Q*^2^_cum_ are positive, indicating that noise or redundant information is removed and the explanatory power of the model is improved. The *R*^2^*Y*_cum_ and *Q*^2^_cum_ values are approximately equal to each other, which implies that the model performed consistently in terms of both interpretability and the predictability. The *Q*^2^ values of the tablet quality prediction models obtained using the material library approach and the latent variable modeling methods presented in the literature were about 0.70–0.80 [49,50,51], which were similar to the model evaluation results of the model in this study.

**Figure 3:**
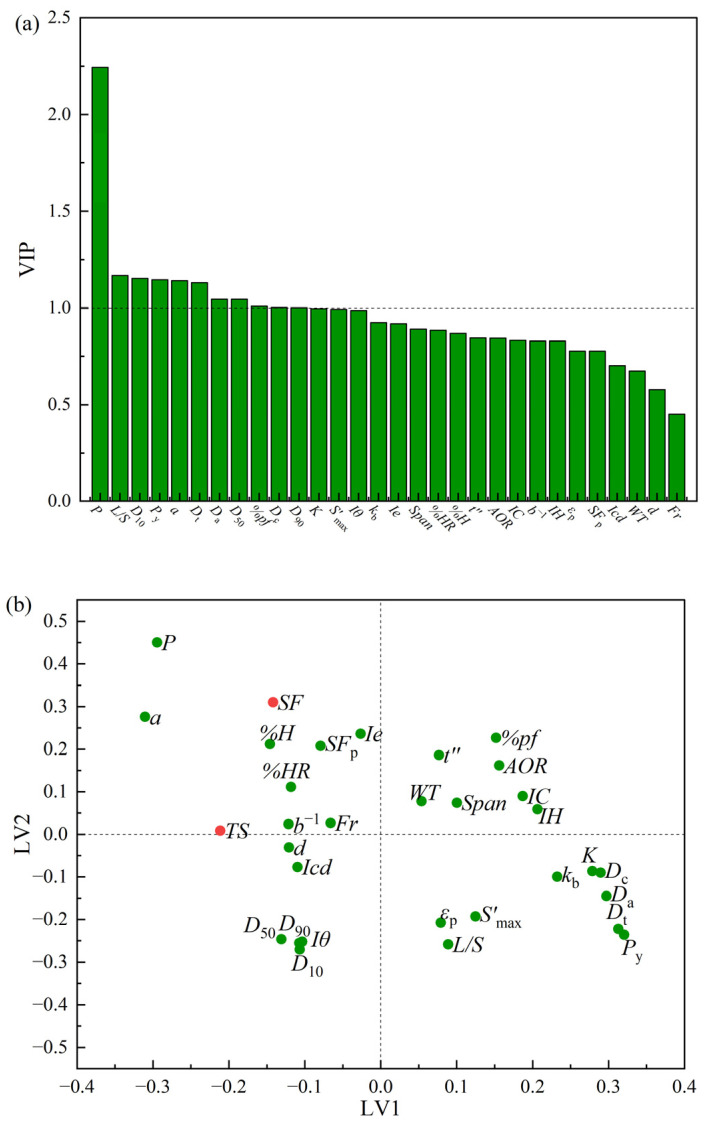
(**a**) The variable importance in the projection plot of PLS Model M2. (**b**) The loading plot of PLS Model M2.

Figure 3a shows the VIP plot of the PLS Model M2. Eleven variables with VIP values greater than 1 were identified as critical. The tableting pressure (*P*) and the *L*/*S* ratio were critical processing parameters; in addition, the properties of powders, including *D*_10_, *P*_y_, *a*, *D*_t_, *D*_a_, *D*_50_, *%pf*, *D*_c_, and *D*_90_, were observed to be critical. The tableting pressure and the *L*/*S* ratio are of greater importance in the determination of tablet quality. The particle size and density parameters of powders, as well as the material’s plasticity, were of interest when designing the formulation. Figure 3b shows the loading plot on the first two LVs of PLS Model M2. It could be observed that the bonding capacity parameter (*k*_b_), the compression resistance parameter (*K*), and the powder density parameters (i.e., *D*_a_, *D*_c_ and *D*_t_) were negatively correlated with *TS,* labeled with a red dot. This indicated that the stronger the bonding ability of the powder, and the less difficult it was to be compressed, the greater the *TS* of the tablet. The tableting pressure (*P*) and the compressibility descriptor (*a*) were positively correlated with *SF*, while the *L*/*S* ratio and the plastic deformation parameter (*P*_y_) were negatively correlated with *SF*. This indicated the higher compression pressure exerted on the tablet and the stronger plastic deformation of the powder were prone to producing tablets with fewer porosities. In addition, increasing the *L*/*S* ratio may promote the agglomeration of granules in terms of enhancing the coalescence and increasing the granule sizes; hence, both the *SF* and *TS* of the tablet decreased. *Fr* and *WT* had low VIP values and were close to the center of the loading plot, implying that they had a weak impact on the tablet quality attributes. It was also suggested that more attention should be paid to adjusting the starting material properties and optimizing the liquid-to-solid ratio and the tableting force during material–process–product integrated design.

### 3.3. Sensitivity Analysis of Process Parameters for Different Types of Materials

**Figure 4:**
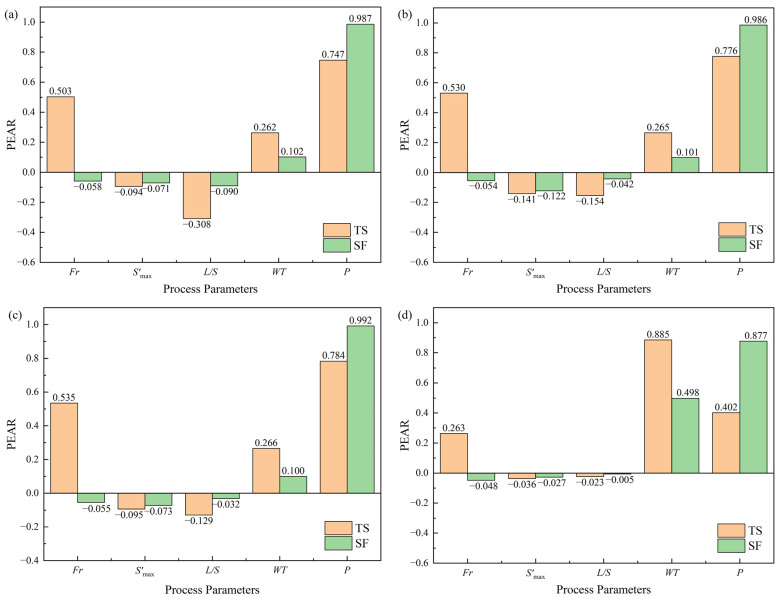
The sensitivity analysis of process parameters for typical materials. (**a**) The silicified microcrystalline cellulose (SMCC). (**b**) The cold-water-insoluble starch (CWIS). (**c**) The Bran-Processed Atractylodis Rhizoma (BPAR). (**d**) The Lactose Flowlac^®^ 100 (Lac F100).

In order to quantify how uncertainty in the process parameters affected the tablet quality, a Monte Carlo-based sensitivity analysis (SA) was carried out for four typical materials, i.e., the plastic material SMCC, the brittle material Lac F100, the starch derivative material CWIS, and the natural product powder BPAR. Five process parameters, the liquid-to-solid ratio, the Frode number, the ${S^{\prime }}_{\max}$, the wet massing time, and the tableting pressure, were varied over specific ranges in Table S3 of Supplementary Material S1, and they were all defined as having uniform distributions. For a specific material, a sample of 1000 vectors was randomly generated from the distribution of the process parameters. Then, each vector of the simulated process parameters (size 1 × 5) was combined with the corresponding material properties vector (size 1 × 25) to create a simulated input matrix (size 1000 × 30). The PLS Model 2 built in Section 3.2 was fed with the simulated input matrix and a set of model outputs was produced. The Pearson product moment correlation coefficient (PEAR), which was the linear correlation coefficient computed between an input variable and an output variable, was used for SA evaluation, since the PLS model is a generalized linear model. The SA results are shown in Figure 4.

An SA evaluation of SMCC is shown in Figure 4a. The SMCC had a good plastic deformation capacity, which was decreased after wet granulation [52]. As the granulation water level or the *L*/*S* ratio increases, the wet granulation may lead to larger granules and lower tabletability [53]. In contrast, Koyanagi et al. [54] found that an increase in impeller speed resulted in an increase in centrifugal force during granule formation, a decrease in granule formation capacity, and an increase in tabletability. In this case, the medium granule size (*G*_50_) values for SMCC under the impeller speeds of 600 rpm, 400 rpm, 800 rpm and 800 rpm from the Runs No. 10 to 13 in Table S2 of Supplementary Material S1, were 187.9 μm, 165.7 μm, 102.2 μm and 106.3 μm, respectively. The corresponding *Span* values were 2.4, 1.9, 1.8 and 1.7, respectively, and the intergranular porosities were 0.27, 0.22, 0.46 and 0.44, respectively. This demonstrated that a higher shear force generated smaller granules with a narrower granule size distribution, as well as higher intergranular porosities, counteracting the granule size-enlargement effect. When the ability to form granules was reduced, the binding surface between the granules could be increased and the *TS* of the tablet was increased [55]. In this paper, the granule size before the tableting process was controlled according to Section 2.3.2, and the size-enlargement effect induced by the *L*/*S* ratio was relatively weakened. As depicted in Figure 4a, the variance of the tablet solid fraction depends negatively on the *L*/*S* ratio. It was found that the measured skeleton densities for SMCC granules under the *L*/*S* ratios of 1.4, 0.8, 0.2, and 0.2 from the Runs No. 10 to 13 in Table S2 of Supplementary Material S1, were 1.55 g∙cm^−3^, 1.48 g∙cm^−3^, 1.62 g∙cm^−3^, and 1.73 g∙cm^−3^, respectively. For SMCC granules, the helium density values decreased as the liquid-to-solid ratios increased. This indicated that a higher *L*/*S* ratio generated more closed pores within granules that could not be detected by the pycnometry technique [56]. Such sealed-off pores might be responsible for the evolution of tablet porosity.

The SA evaluations of CWIS and BPAR, as shown in Figure 4b,c, respectively, are similar to that for SMCC. However, the CWIS and BPAR materials were less sensitive to the *L*/*S* ratio in comparison with the SMCC. The reasons for this may be that both CWIS and BPAR used high concentrations of alcohols as the wetting agent, which had low viscosity and surface tension [57]. The variance in the BPAR tablet tensile strength is negatively correlated with the *L*/*S* ratio, as shown in Figure 4c, which was mainly due to the granule size-enlargement effect. As the *L*/*S* ratios increased from 0.162 (e.g., Runs No. 2) to 0.4455 (e.g., Runs No. 1), the *G*_50_ values of BPAR granules increased from 23.0 μm to 75.4 μm, showing a significant granule growth. The variance in the BPAR tablet solid fraction is also negatively correlated with the *L*/*S* ratio. The pycnometry densities for BPAR granules under the *L*/*S* ratios of 0.4455 and 0.162 from Runs No. 1 and No. 2 in Table S2 of Supplementary Material S1 were 1.37 g∙cm^−3^ and 1.22 g∙cm^−3^, respectively. This meant that a higher *L*/*S* ratio generated more dense granules, which is different from results obtained for the SMCC material.

Figure 4d shows the SA plot for the Lac F100. Another brittle material, i.e., the DCP, had a similar SA plot, which is not shown here. It could be observed that the effects of process parameters like the *L*/*S* ratio and ${S^{\prime }}_{\max}$ on the tablet quality attributes of lactose were negligible, because the granule size enlargement could be compensated by the extensive rearrangement and fragmentation of granules [58]. The shear force may promote brittle fracture during wet granulation, which increases the bonding area and the ability of granules to interact with each other.

Overall, from the sensitivity analysis, it was demonstrated that the granulation and tableting knowledge for different materials was understood by the process model. The sensitivity analyses can rank process variables by the effect they have on output variables. It was found that the agitation intensity could compensate for the granule size enlargement which negatively influenced tablet tensile strength. In addition, the tablet porosity may be affected by the intragranular or intergranular porosity, which was material-specific and needs to be further verified.

### 3.4. The Integrated Design of Formulation and Process Parameters

#### 3.4.1. The Integrated Design Method

The ideal mixing rule [59] and the tablet quality attributes prediction model developed in Section 3.2 were integrated to support the simulation of the formulation design and process design. The predicted mixture properties served as interconnecting variables, as well as input of the tablet quality attributes prediction model. The following four steps detail how to realize formulation–process–product integrated design.

Step 1: Target Definition and Material Characterization

The target tensile strength of a tablet was at least 2.0 MPa and the acceptable solid fraction was 0.80–0.90. Given a drug material to be formulated, the physical properties and CBCS descriptors were characterized according to Section 2.4.

Step 2: Formulation Simulation

In Step 2, the formulation simulation could be used to fully explore different combinations of drug and excipients. The number of candidate excipients was denoted as *s*. The material property parameters of the candidate excipients was characterized in the same way as those of drugs or could be retrieved from the iTCM material database. The weight percent of all excipients in a tablet formulation was denoted as *m*, and the weight percent of drug was 1 − *m*. A vector **a** = [*a*_1_, *a*_2_,…, *a*_i_] was randomly generated, where *a*_1_ + *a*_2_ + … + *a*_i_ = *m*, and *i* ∈ 1, 2, …, *s*. The number of randomized simulations was set to *b* and a ratio matrix **O** (size *b* × *s*) was obtained. In order to control the complexity of the tablet ingredients, the maximum number of excipients in the designed tablet was set to 5. Therefore, the row vectors in matrix **O** with none-zero scalars less than or equal to 5 were screened out to form a new matrix **E** (size *c* × *s*), where *c* was the number of satisfied row vectors. After that, the formulation matrix **A** was constructed by combining a column vector **D** (size *c* × 1) and the matrix **E**, and each scalar in column vector **D** was the weight percent of drug 1 − *m*. The size of **A** was *c* × (1 + *s*), and each row of **A** represented a candidate tablet recipe.

Furthermore, the material property matrix **I** of the drug and excipient candidates was built. The size of **I** was (1 + *s*) × *q*. The first row of the matrix **I** represented *q* material properties of the drug (in M2, *q* = 25). Based on the ideal mixing rule, the simulated formulation mixture property matrix **AI** was calculated as **AI** = **A** × **I**, and the size of **AI** was *c* × *q*.

Step 3: Process Simulation

Consistent with the PLS Model M2, five process parameters, i.e., *Fr*, *L*/*S*, ${S^{\prime }}_{\max}$, *WT*, and *P*, were varied to explore the process space of HSWGT. *Fr* denotes the agitation intensity. For a given high-shear wet granulator with a different scale but geometric similarity, the impeller speed can be inferred from the *Fr* formula. The wetting agent and the *L*/*S* ratio were specific to certain materials. In the design stage, a PCA-based material properties matching model was used to determine the suitable wetting agent and the *L*/*S* ratio for a newly designed formulation. Correspondingly, the range of ${S^{\prime }}_{\max}$ could be estimated according to Equation (2a).

The development procedures of the PCA model and the matching methods are described below. First, the material properties of 30 powdered materials in the primary material library, as depicted in Section 2.1, were used to construct a material dataset, in which each material featured 19 physical property parameters and 9 CBCS descriptors. Then, a PCA of this material dataset was conducted, and the first three PCs explained 66.4% of the total variation. The fourth PC contained limited information, explaining only 8.65% of the total variation. Hence, the PCA model with the first three PCs was chosen for the wetting agent matching. Based on the PCA model, the centroid of powders using water as the wetting agent and the centroid of powders using alcohol as the wetting agent were located. For a simulated tablet formulation (e.g., row *j* of matrix **A** and *j* ∈ 1, 2, …, *c*) in Step 2, the calculated formulation mixture properties (e.g., row *j* of matrix **AI**) were projected onto the PCA model. The Euclidean distances between the simulated tablet formulation and the two centroid points were computed. The centroid connected to the short Euclidean distance was used to designate the application of the same wetting agent for the simulated tablet formulation. After that, the Euclidean distance between each powder in the PCA model and the simulated tablet formulation was calculated. The range of the *L*/*S* ratios for the simulated tablet formulation was designated to be the same as the powder with the shortest Euclidean distance.

Considering the operating convenience of the subsequent experimental validation, the levels for each process parameter of the HSWG and the tableting pressure could be set according to practical situations. For each simulated tablet formulation, one level was randomly selected for a particular process parameter, generating a process parameter matrix **R** (size *c* × 5). Matrices **AI** and **R** were concatenated to obtain matrix **AIR** (size *c* × 30), which was used as input data for the PLS Model M2.

Step 4: Solution Generation

The output of the PLS Model M2 was the predicted tablet tensile strength and solid fraction. The *SPE* less than the *SPE* limit at the significance level 0.05, as well as the Hotelling T^2^ less than the Hotelling T^2^ limit at the confidence level 0.95, were used as constraints. Based on the targets of tablets defined in Step 1, as well as the constraints, the combinations of simulated formulations and process parameters that met the requirements were selected and could be experimentally validated.

#### 3.4.2. Case Study

The FPPID implementation for a tablet containing MHH powders was used as an example. The drug loading capacity of 70 (i.e., m = 30%) was adopted to minimize the administration of excipients. From the material characterization results presented in Step 1, it was found that the *TS* of the MHH powders was less than 2.0 MPa at compression pressures of 1–10 kN. This suggested that the MHH materials had poor tabletability. Fifteen candidate excipients were chosen from the iTCM material library, comprising thirteen diluents (i.e., three types of MCC; three types of lactose, DCP, mannitol, dextrin, corn starch, maltodextrin, CWIS, and β-CD) and two disintegrants (i.e., PPVP and CMC-Na). The ratio matrix **O** (size 2000 × 15) was generated to find formulations with a number of excipients less than or equal to 5. As a result, a new matrix **E** (size 1566 × 15) was obtained. The simulated formulation matrix **A** (size 1566 × 16) was created according to Step 2. Using the ideal mixing rule, the simulated formulation mixture property matrix **AI** (size 1566 × 25) was then estimated. In Step 3, the simulation ranges for the Frode number, the wet massing time, and the tableting pressure were 0.63–2.54, 180 s–300 s, and 1 kN–11 kN, respectively. The range of the Frode number was translated into the range of the impeller speed (i.e., 400 rpm–800 rpm) to facilitate the easy manipulation of the HSWG process.

**Figure 5:**
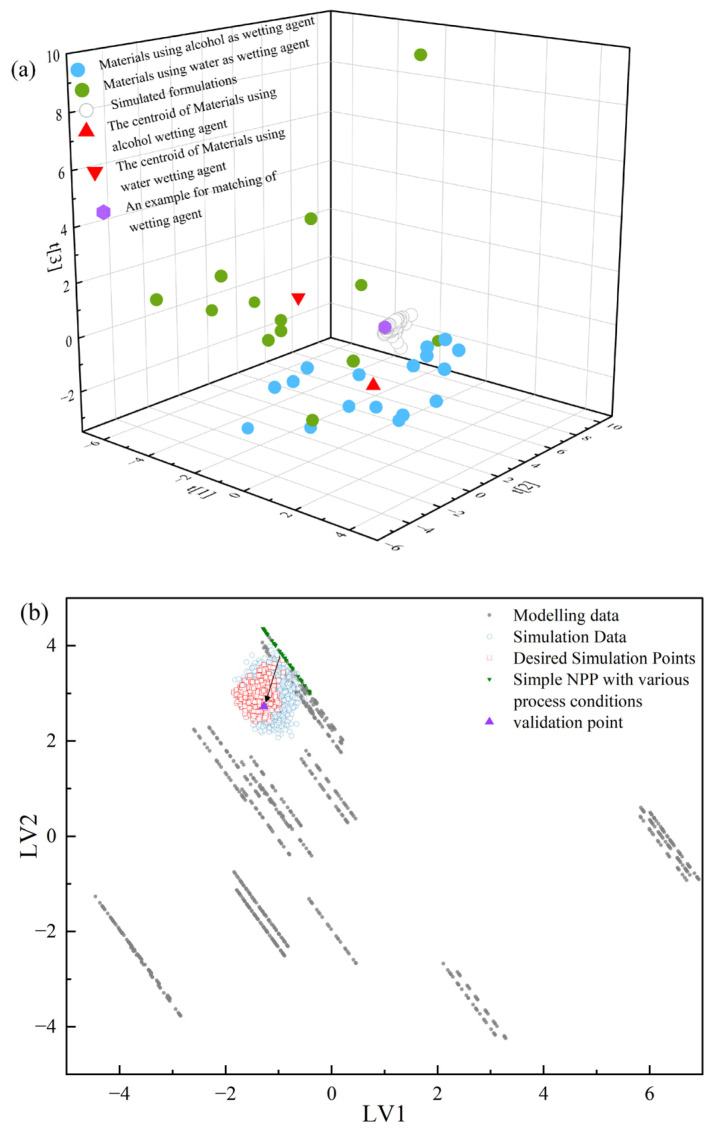
(**a**) The matching of the wetting agent for simulated formulations. (**b**) Visualization of the solution generation process in the score plot of PLS Model M2.

The wetting agent was determined using the PCA-based material properties matching method. Taking the simulated formulation in Row No. 1 of **A** as an example, the estimated material properties vector is projected onto the PCA score space, as shown by the purple hexagon in Figure 5a. The centroids of powders using alcohol and water as the wetting agents are represented by the red triangle dot and the red inverted triangle dot, respectively. The Euclidean distance from the purple hexagon dot to the centroid of powders using a water wetting agent was 4.30, and the Euclidean distance between the purple hexagon dot and the centroid of powders using an alcohol wetting agent was 2.09. Therefore, the 95% alcohol was selected as the wetting agent for the No. 1 formulation. The purple hexagon dot was nearest to the BPAR powder and the *L*/*S* ratio 0.16–0.72 was used for BPAR. The range of ${S^{\prime }}_{\max}$ was calculated to be 0.05–0.23. Furthermore, all simulated formulations were projected onto the score space, as shown by the hollow gray dots in Figure 5a. It can be seen that they were all close to the BPAR powder, and the same *L*/*S* ratio was therefore applied in subsequent simulations.

In Figure 5a, the blue dots represent the materials using alcohol as a wetting agent. The green dots represent the materials using deionized water as a wetting agent. The gray hollow dots represent the simulated formulations. The red positive triangle represents the centroid of the materials using an alcohol wetting agent. The red inverted triangle represents the centroid of the materials using a water wetting agent. The purple hexagon is an example formulation. In Figure 5b, the gray dots represent the data points used for building the model. The blue hollow dots represent the simulated combinations of the formulation and process conditions. The red hollow squares represent the desired simulation points that meet the objectives specified in Step 4 of FPPID. The green inverted triangles represent a single NPP material under different process conditions. The purple positive triangle represents the selected validation point.

For convenient operation, the impeller speed varied between 400 rpm and 800 rpm at intervals of 100 rpm. The batch amount was about 300.0 g. The amount of wetting agent to be added was calculated using the *L*/*S* ratio, and changed from 30 mL to 240 mL at intervals of 30 mL. The wet massing time varied from 180 s to 300 s at intervals of 30 s. The tableting force varied from 1 kN to 11 kN at intervals of 2 kN. The process parameter matrix **R** (1566 × 5) was produced by randomly assigning a level to each process parameter. After that, the concatenated matrix **AIR** (1566 × 30) was generated and fed into the PLS Model M2.

Figure 5b shows the two-dimensional latent variable space of PLS Model M2. The gray dots are the 735 data points used to build the PLS Model. The blue hollow dots are 1566 simulated data points. The target range of the *SF* could be slightly wider than that defined by the MCS group (i.e., 0.80–0.90), since the NPPs were soluble in water and the disintegration of high-drug-load NPP tablets was less impacted by the tablet’s porosity [60]. In this case, combined with the RMSE for SF, the target range of SF can be modified to 0.705–0.995. The SPE limit at the significance level 0.05 was 3.84. The Hotelling T^2^ limit at the confidence level 0.95 was 12.75. According to the target *TS* and *SF*, as well as the constraints for the Hotelling T^2^ and SPE, 119 solution points were screened out and are represented as red hollow dots in Figure 5b. The green inverted triangles are the projections of the pure NPP materials under different tableting pressures. The purple triangle represents the validation points randomly selected from the solution points. A total of 300 g of powder was weighed according to the proportion of the predicted formulation, which was mixed using a three-dimensional mixer (ZNW-10, Beijing Xingshi Lihe Science and Technology Development Co., Ltd., Beijing, China) at a rotational speed of 14 r∙min^−1^ for 15 min. Then, according to the process parameters in the validation plan, the granulation and tableting were carried out according to the preparation methods in Section 2.3. The actual *TS* and *SF* were measured and compared with the predicted values. The specific data of the matrix and solution points in the validation example cases are presented in Supplementary Material S2. As shown in Table 5, the validation formulation fulfilled the predefined targets in Step 1. This demonstrated the effectiveness of the proposed FPPID method.

The FPPID implementation for MHH-2 was the same as that in the MHH-1 case. The design and validation results are shown in Table 5. It could be seen that the designed tablets satisfied the targets in Step 1. In the validation experiment of MHH-1, the predicted results show that *TS* is 2.18 MPa and *SF* is 0.94. The experimental results are *TS* = 2.03 MPa; *SF* = 0.89. In the validation experiment of MHH-2, the predicted results are *TS* = 3.01 MPa; *SF* = 0.97. The experimental results are *TS* = 2.00 MPa; *SF* = 0.84. These are within the acceptable range, and the model has a good ability to generalize. In addition to this, the disintegration time and dosage uniformity of the predicted formulations were examined to help validate the applicability of the model. According to the US Pharmacopoeia 35 <705>, one dosage unit was placed in each of the six test tubes of the disintegration basket. The deionized water was used as the immersion fluid, maintained at 37 ± 2 °C, and the round-trip frequency was set at 30 times/min. The disintegration time of MHH-1 was 10.20 min, and that of MHH-2 was 8.48 min. Since the content of *API* in the validation experiment was more than 25% of the total tablet weight, the weight variation method was chosen for the experiments to ensure uniformity of the dosage. In each experiment, ten tablets were randomly selected and precision-weighed, and the mean tablet weight and the relative standard deviation (*RSD*) of the 10 tablets were calculated. The average weight of tablets in MHH-1 was 0.3369 g, and the average weight in MHH-2 was 0.3428 g. The *RSD* was 0.28% for MHH-1 and 0.16% for MHH-2. The two sets of validated formulations fulfilled the requirements of the examination of dosage uniformity. In the future, it is recommended that more appropriate formulations be selected by looking at the crucial parameters of candidate pharmaceutical excipients in order to generate more reliable solutions.

## 4. Conclusions

In this paper, a collective application of the available design knowledge to speed up pharmaceutical tablet development through the HSWGT route was successfully realized using the proposed formulation–process–product integrated design framework. Several design elements proved to be effective in supporting the FFPID methodology. First, the material library provided an information-rich material property space, enhancing the model’s prediction and generalization capability as well as ensuring a sufficiently large digital domain for candidate formulation materials selection. Secondly, the latent variable model quantified the relationships between the input material properties, the HSWGT processes, and the tablet quality attributes. The granulation and tableting knowledge for different materials were stored in a unified and formalized manner. The dimensionless parameters from the wet granulation regime map approach were incorporated into the data-driven process models, leaving room for model extrapolation beyond the variables manipulated at the experimental scale. In addition, the mixture properties calculation model and the process model were combined together, and the interconnected model chains enabled the simultaneous exploration of the formulation design space and the process design space. Furthermore, a four-step model-driven design methodology, including the target definition, the formulation simulation, the process simulation, and the optimization, was established to streamline the multi-step tablet development process. Finally, the performance of the proposed FPPID framework was demonstrated through the efficient development of high-drug-loading tablets.

With the increasing application of digital technology in the pharmaceutical development, the extensively used QbD paradigm gradually evolved into quality by digital design (QbDD), which is a computational model-aided enhancement of the QbD framework. The MDD or digital design emphasized the utilization of the mathematical model and in silico simulation and possessed the features inherent to QbDD. The FPPID method presented in this paper incorporates the material database, integrated design models, design space visualization, and reliability-based decision metrics, and an example of its implementation QbDD in oral solid dosage development is presented. The FPPID offers great opportunity for designers to handle the complex interplay in the sequence of development stages, facilitate instant and informed decisions, and accelerate product development. Regarding the FPPID framework built for the HSWGT, its deductive capabilities regarding the binder selection and the optimization of the drying and the milling stages could be strengthened using data-driven approaches in the future. Moreover, extending the FPPID framework to other OSD-processing routes is also the focus of future research.

## Figures and Tables

**Table 1:** The designed factors and levels for the high-shear wet granulation process.

Factors	Symbols	Category	Coded and Actual Values in Experiments		
			Low (−1)	Medium (0)	High (1)
Type of material	*Z*	Block	/	/	/
*L*/*S* ratio (g·g^−1^)	*X*_1_	Categorical	Level 1	Level 2	Level 3
The impeller speed (rpm)	*X*_2_	Continuous	400	600	800
The wet massing time (s)	*X*_3_	Continuous	180	240	300

**Table 2:** The physical parameters for characterizing powders.

Incidence Factor	Parameters	Acronyms
Fundamental property	True density	*D*_t_
	Particle size	*D*_10_, *D*_50_, *D*_90_
Dimension	Bulk density	*D*_a_
	Tapped density	*D*_c_
	Porosity	$\varepsilon_{p}$
	Solid fraction of the powder	*SF*_p_
Compressibility	Inter-particle porosity	*Ie*
	Carr’s index	*IC*
	Cohesion index	*Icd*
Flowability	Hausner ratio	*IH*
	Angle of repose	*AOR*
	Flow time	*t*″
Stability	Moisture content	*%HR*
	Hygroscopicity	*%H*
Homogeneity	Particle size less than 50 μm	*%pf*
	Homogeneity index	*Iθ*
	Particle size distribution	*Span*

**Table 3:** The compression descriptors from the compression behavior classification system.

The Compression Behavior	The Name of Equation	The Equation	The Curve-Fitting Relationship	Compression Descriptors	The Meaning of Descriptors
Compressibility	The Kawakita equation	$\frac{P}{C}=\frac{1}{\mathrm{ab}}+\frac{P}{a}$	Porosity-*P*	*a*, *b*^−1^, *ab*	*a* denotes the degree of compression of the powder at maximum pressure; *b*^−1^ denotes the pressure required to reach a/2; *ab* denotes the rearrangement capacity of the particles.
	The Heckel equation	$\mathrm{Ln}(\frac{1}{\varepsilon })=\mathrm{KP}+A$ *P*_y_ = $\;\frac{1}{K}$		*P*_y_	Powder plasticity
	The Shapiro equation	$\mathrm{Ln}(\varepsilon )=\ln\varepsilon_{0}-\mathrm{kP}-fP^{0.5}$		f	Degree of particle crushing, brittleness
	The Gurnham equation	$\varepsilon =-\frac{1}{K}\ln(\frac{P}{P_{0}})$		*K*	Powder compression resistance
Compactibility	The R-D equation	$\mathrm{TS}={\mathrm{TS}}_{0}\exp(-K_{b}\varepsilon$)	Porosity-*TS*	*k*_b_	Interparticle binding ability
Tabletability	The power equation	$\mathrm{TS}=dP^{g}$	*TS*-*P*	*d*, *g*	*d* denotes tabletability; *g* indicates pressure sensitivity

**Table 4:** The input and output variables of the PLS model for the high-shear wet granulation and tableting process.

The Type of Variable		The Matrix Size	The Number of Variables	The Variables
Input variable	The powder physical properties	735 × 33	19	*D*_10_, *D*_50_, *D*_90_, *Span*, *%pf*, *Iθ*, *D*_a_, *D*_c_, *D*_t_, *SF*_p_, *ε*_p_, *IH*, *IC*, *Ie*, *t″*, *AOR*, *%H*, *%HR*, *Icd*
	The compression behavior descriptors		9	*a*, *b*^−1^, *ab*, *f*, *P*_y_, *K*, *k*_b_, *d*, *g*
	The HSWG process parameters		4	*L*/*S*, *WT*, *Fr*, ${S^{\prime }}_{\max}$
	The tableting pressure		1	*P*
Output variable	Tablet attributes	735 × 2	2	*TS*, *SF*

**Table 5:** Validation of optimal design solutions.

Run No.	Drug Material	Designed Formulation (*w*/*w*)	Designed Process Parameters	Predicted Quality Attributes	Validated Quality Attributes
1	The MHH extract powder	70% MHH, 26% MCC PH101, 2% Mannitol, 1% DCP, 1% Lac C80	Wetting agent: 95% ethanol, *L*/*S* ratio: 0.243, *Fr*: 0.63, ${S^{\prime }}_{\max}$: 0.05, *WT*: 270 s, *P*: 7 kN	*TS* = 2.18 MPa *SF* = 0.94	*TS* = 2.03 MPa *SF* = 0.89
2	The MHH extract powder	70% MHH, 21% MCC PH101, 1% Lac F100, 4% Mannitol, 4% DCP	Wetting agent: 95% ethanol, *L*/*S* ratio: 0.243, *Fr*: 0.99, ${S^{\prime }}_{\max}$: 0.048, *WT*: 300 s, *P*: 9 kN	*TS* = 3.01 MPa *SF* = 0.97	*TS* = 2.00 MPa *SF* = 0.84

## Data Availability

The data presented in this study are available in Supplementary Materials.

## Supplementary Materials

The following supporting information can be downloaded at: https://www.mdpi.com/article/10.3390/pharmaceutics17030322/s1. Supplementary Materials S1: Table S1: The experimental design for the high shear wet granulation and tableting process; Table S2: The physical properties and compression behavior desciptors of powders and granule; Table S3:The designed factors and levels for the high shear wet granulation process in sensitivity analysis. Supplementary Materials S2: Tables S1–S6: Case study data.

## Abbreviations

API	Active pharmaceutical ingredient
β-CD	β-cyclodextrin
BPAR	Bran-processed atractylodis rhizoma
CBCS	Compression behavior classification system
CDER	Center for Drug Evaluation and Research
CMC-Na	Croscarmellose sodium
CWIS	Cold water insoluble starch
DC	Direct compression
DCP	Dibasic calcium phosphate
DCPA	Dibasic calcium phosphate anhydrous
DG	Dry granulation
FDA	US Food and Drug Administration
FPPID	Formulation–process–product integrated design
*Fr*	Froude number
*G*_50_	Medium granule size
HSWG	High-shear wet granulation
HSWGT	High-shear wet granulation and tableting
*L*/*S*	Liquid to solid
Lac F100	Lactose Flowlac^®^ 100
MBD	Model-based design
MCS	Manufacturing classification system
MDD	Model-driven design
MF	Mume fructus
MHH	Menthae haplocalycis herba
NPP	Natural product powder
OSD	Oral solid dosage
*P*	Tableting pressure
PC	Principal component
PCA	Principal component analysis
PEAR	Pearson product moment correlation coefficient
PLS	Partial least square
QbD	Quality by design
QbDD	Quality by digital design
R^2^	Coefficient of determinism
RMSE	Root mean square error
RMSECV	Root mean square error of cross-validation
RSD	Relative standard deviation
SA	Sensitivity analysis
SF	Solid fraction
${S^{\prime }}_{\max}$	Maximum pore saturation
SMCC	Silicified microcrystalline cellulose
SPE	Square prediction error
TS	Tensile strength
TSWG	Twin screw wet granulation
UV	Unit variance
LV	Latent variable
VIP	Variable importance in projection
WG	Wet granulation
WT	Wet massing time
